# The neurobiological mechanism underlying hypothalamic GnRH pulse generation: the role of kisspeptin neurons in the arcuate nucleus

**DOI:** 10.12688/f1000research.18356.2

**Published:** 2020-02-06

**Authors:** Tony M. Plant

**Affiliations:** 1 Magee-Womens Research Institute, University of Pittsburgh, 204 Craft Avenue, Pittsburgh, PA 15213, USA

**Keywords:** Kisspeptin, neurokinin B, dynorphin, GnRH, pulse generation

## Abstract

This review recounts the origins and development of the concept of the hypothalamic gonadotropin-releasing hormone (GnRH) pulse generator. It starts in the late 1960s when striking rhythmic episodes of luteinizing hormone secretion, as reflected by circulating concentrations of this gonadotropin, were first observed in monkeys and ends in the present day. It is currently an exciting time witnessing the application, primarily to the mouse, of contemporary neurobiological approaches to delineate the mechanisms whereby *Kiss1/NKB/Dyn* (KNDy) neurons in the arcuate nucleus of the hypothalamus generate and time the pulsatile output of kisspeptin from their terminals in the median eminence that in turn dictates intermittent GnRH release and entry of this decapeptide into the primary plexus of the hypophysial portal circulation. The review concludes with an examination of questions that remain to be addressed.

## Introduction

The intermittent or pulsatile release of gonadotropin-releasing hormone (GnRH) from the hypothalamus into the hypophysial portal circulation is obligatory for driving gonadotropin secretion from the anterior pituitary and therefore for gonadal development and maturation, puberty, and fertility in adulthood ^1^. The intermittent pattern of GnRH release is imposed upon the diffusely distributed hypothalamic GnRH neurons by a neural network termed the GnRH pulse generator. Realization of the existence of such a pulse-generating mechanism in the hypothalamus may be traced to 1970. However, it was not until 2003, following the discovery that loss-of-function mutations of the gene encoding the kisspeptin receptor (KISS1R), a G protein–coupled receptor (GPR, specifically GPR54), were associated with delayed or absent puberty that major advances in our understanding of the neurobiological components of the GnRH pulse generator began to emerge. Moreover, another decade passed before the contemporary view that kisspeptin represents the output of the GnRH pulse generator was proposed. The purpose of this article is to review the signal developments at both the empirical and conceptual levels that have led, over half a century of scientific endeavor, to our present understanding of how hypothalamic GnRH pulses are generated by kisspeptin neurons in the arcuate nucleus.

## The early years

In attempting to resolve whether the marked differences in daily concentrations of circulating luteinizing hormone (LH) observed in ovariectomized monkeys were the consequence of physiology or assay error, Knobil’s group in the late 1960s measured LH levels in sequential plasma samples collected every 10 or 20 minutes from such animals ^2^ ^I^. The group reported a striking sawtooth pattern in plasma LH concentrations. Although unstable levels of circulating LH levels had been revealed by hourly sampling of the castrate male rat ^3^, it was Knobil’s laboratory that correctly posited that the discrete, roughly hourly episodes of LH secretion that produced the pulses in the circulating gonadotropin were due to intermittent signals from the brain that were relayed to the anterior pituitary by an LH-releasing factor. This was in the year before the structure of the LH-releasing factor (the decapeptide now termed GnRH) was finally reported ^4, 5^, and it was another 10 years before Clarke and Cummins ^6^ described the temporal correlation between discharges of GnRH in portal blood and LH pulses in the peripheral circulation, thereby providing empirical confirmation for the hypothesis proposed by Dierschke *et al*. ^2^ ^II^.

That the intermittent signal generating GnRH pulses originated within the mediobasal hypothalamus (MBH) was suggested by the finding that, in ovariectomized female rats and monkeys, pulsatile LH secretion was not interrupted by surgical isolation of the MBH ^9, 10^. Knobil’s group later reported, again in the ovariectomized rhesus monkey, that discrete bilateral radiofrequency lesions of the arcuate nucleus at the base of the third ventricle (also known as infundibular nucleus in humans) abolished LH secretion but that larger lesions of the MBH that spared this nucleus were noticeably less disruptive ^11^. These findings suggested that the arcuate nucleus, which is located immediately dorsal to the primary plexus of the hypophysial portal circulation, was essential for generating the intermittent GnRH signals driving pulsatile LH release.

## Evolution of the concept of the hypothalamic GnRH pulse generator

**Figure 1.**
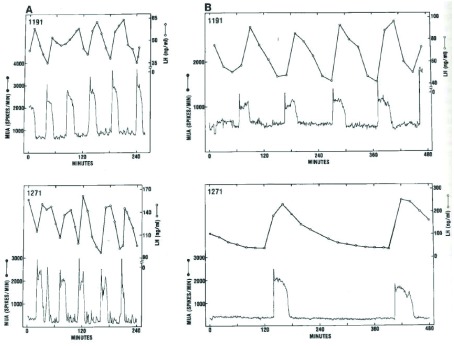
Pulsatile luteinizing hormone secretion is tightly correlated with electrophysiological activity in the monkey mediobasal hypothalamus. The relationship between luteinizing hormone (LH) “pulses”, observed in the peripheral circulation (open data points), and multiunit activity (MUA) (continuous trace) recorded in the mediobasal hypothalamus of two ovariectomized rhesus monkeys before (Panel **A**) and after (Panel **B**) anesthesia was induced with thiopental. Note the high-fidelity relationship between the two parameters regardless of frequency of pulsatile LH secretion. Reproduced from Wilson *et al*. ^16^ with permission of Karger Publishers. Copyright © (1984) Karger Publishers, Basel, Switzerland.

Although the “black box” concept of a hypothalamic pulse generator or arcuate oscillator driving pulsatile GnRH and LH secretion appeared in the literature in the early 1980s ^12, 13^, it was not until the recognition that pulsatile LH secretion was tightly correlated with electrical activity in the MBH recorded with multiunit electrodes (multiunit activity, MUA) ( Figure 1) ^14– 16^ ^III^ that the concept of the hypothalamic GnRH pulse generator became a cornerstone of the control system governing the reproductive axis. One school of thought argued that pulsatility was intrinsic to the GnRH neurons themselves whereas another school proposed that this mode of secretion was imposed on the network of GnRH neurons by non-GnRH cells in the MBH ^17^. Evidence for the former idea was provided by the finding that immortalized GnRH neurons in culture produced a discontinuous pattern of release of GnRH into the media ^18^ reminiscent of the pulsatile mode of GnRH secretion *in vivo*. However, the neurobiological mechanisms that underpin GnRH release *in vitro* are not operative *in vivo* ^17^. Compelling support for extrinsic pulse generation was provided by two studies conducted in the 1990s. Ohkura *et al*. ^19^ reported that the acute suppression of LH pulsatility, which was produced in ovariectomized rats following a surgical 180° hypothalamic deafferentation and which interrupted anterior inputs (including those from GnRH cell bodies) to the arcuate nucleus at its rostral aspect, was prevented by prior transplantation at the base of the third ventricle of fetal MBH devoid of GnRH neurons. Purnelle *et al*. ^20^ subsequently demonstrated that retrochiasmatic hypothalamic explants from the rat that contained the arcuate nucleus and GnRH fiber projections to the median eminence, but not GnRH cell bodies, exhibited pulsatile GnRH release in culture.

**Figure 2.**
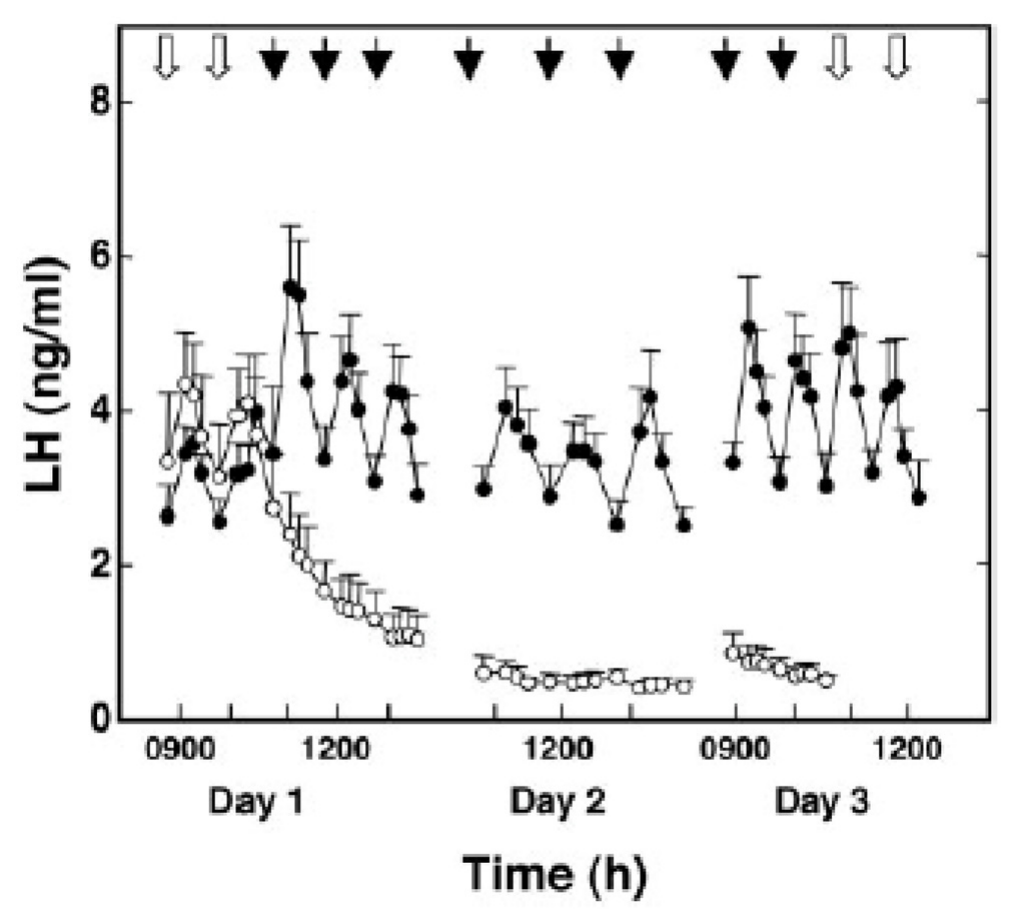
Intermittent kisspeptin administration drives a corresponding pattern of GnRH dependent luteinizing hormone discharges in monkey. Intermittent intravenous administration of kisspeptin-10 (2 μg/min for 1 min/hour starting at 11 a.m. on day 1 and continuing for 48 hours, closed data points) induces a sustained train of luteinizing hormone (LH) “pulses” in a naturally GnRH-deficient primate model (juvenile male rhesus monkey) that matches that generated by an antecedent intermittent GnRH infusion, also administered at 1 pulse/hour before kisspeptin administration (9 to 11 a.m., day 1). The LH response to kisspeptin was abolished by prior treatment with a GnRH receptor antagonist (not shown). Results for vehicle are shown in the open data points. Although kisspeptin-10 or vehicle was administered every hour for 48 hours, LH responses were tracked for only two or three pulses per day. Black arrows indicate times of pulse infusions of kisspeptin-10 or vehicle that were selected for monitoring the LH response. White arrows indicate time of GnRH pulse infusions. Values are presented as mean ± standard error. GnRH, gonadotropin-releasing hormone. Reproduced from Plant *et al*. ^32^ with permission of the Endocrine Society.

The first real insight into this black box came as a result of the 2003 discovery that humans and mice with loss-of-function mutations of *KISS1R* were hypogonadotropic and had delayed or absent puberty ^24, 25^. Moreover, in addition to the very low circulating LH levels associated with the mutated gene, secretion of LH in human subjects bearing the mutation could be induced by intermittent exogenous GnRH administration ^25^, suggesting that the impact of the mutation was manifest at a supra-pituitary level. Shortly thereafter, expression of *KISS1R* by GnRH neurons was identified in mouse by *in situ* hybridization ^26, 27^, and kisspeptin, the endogenous ligand of KISS1R, was demonstrated to elicit (1) an increase of GnRH levels in the cerebrospinal fluid of sheep ^28^; (2) tetrodotoxin-independent depolarization of green fluorescent protein (GFP)-expressing GnRH neurons in perfused slices of hypothalamus from transgenic mice ^27^ and release of GnRH from mouse MBH explants in culture ^29^, suggesting a major site of kisspeptin action at the level of both the GnRH perikaryon and nerve terminal in the median eminence; and (3) robust and GnRH-dependent increases of circulating LH concentrations in both rodent and primate species ^26, 30, 31^. Moreover, repetitive hourly intravenous (IV) administration of kisspeptin for 48 hours in the juvenile male monkey, in which kisspeptin content of the arcuate nucleus/median eminence is low and hypothalamic GnRH release is profoundly restrained, evokes—when the pituitary is first primed with pulsatile GnRH—a sustained train of GnRH-dependent LH pulses that mimics that observed spontaneously in adult animals with unrestrained GnRH release ( Figure 2) ^32^. Intermittent release of kisspeptin in the region of the arcuate nucleus and median eminence of the ovariectomized monkey was observed and about 75% of these events were associated with, or immediately preceded by, a GnRH discharge ^33^, whereas administration of a KISS1R antagonist directly to this hypothalamic area of the monkey resulted in suppression of GnRH release ^34^. Not surprisingly, mice null for *Kiss1* exhibited a phenotype similar to that of the receptor knockout, and the LH-releasing action of kisspeptin was preserved in these transgenic animals ^35^.

Taken together, the above findings led to the notions that the GnRH pulse generator was extrinsic to the GnRH neuron and that kisspeptin was likely a key component of the black box. These ideas were reinforced by several later findings. Kisspeptin was shown to induce GnRH release into the hypophysial portal circulation in sheep ^36, 37^, and transgenic approaches demonstrated that LH pulsatility was absent in *Kiss1R*-null mice ^38^ and *Kiss1*-null rats ^39^. Most recently, LH pulsatility was restored in the former animal model by selective rescue of expression of the kisspeptin receptor in GnRH neurons ^40^.

## Kisspeptin neurons in the arcuate nucleus provide the output of the hypothalamic GnRH pulse generator

Attention to the arcuate nucleus as the likely site of the hypothalamic GnRH pulse generator was reignited when it was demonstrated that in several species the major site of *Kiss1*/ *KISS1*-expressing and kisspeptin-containing neurons within the MBH resided in this nucleus ^29, 30, 41– 44^. Indeed, the lesions earlier placed in the arcuate nucleus of the monkey that produced a profound decline in gonadotropin secretion ^11^ would have destroyed most of the kisspeptin perikarya.

**Figure 3.**
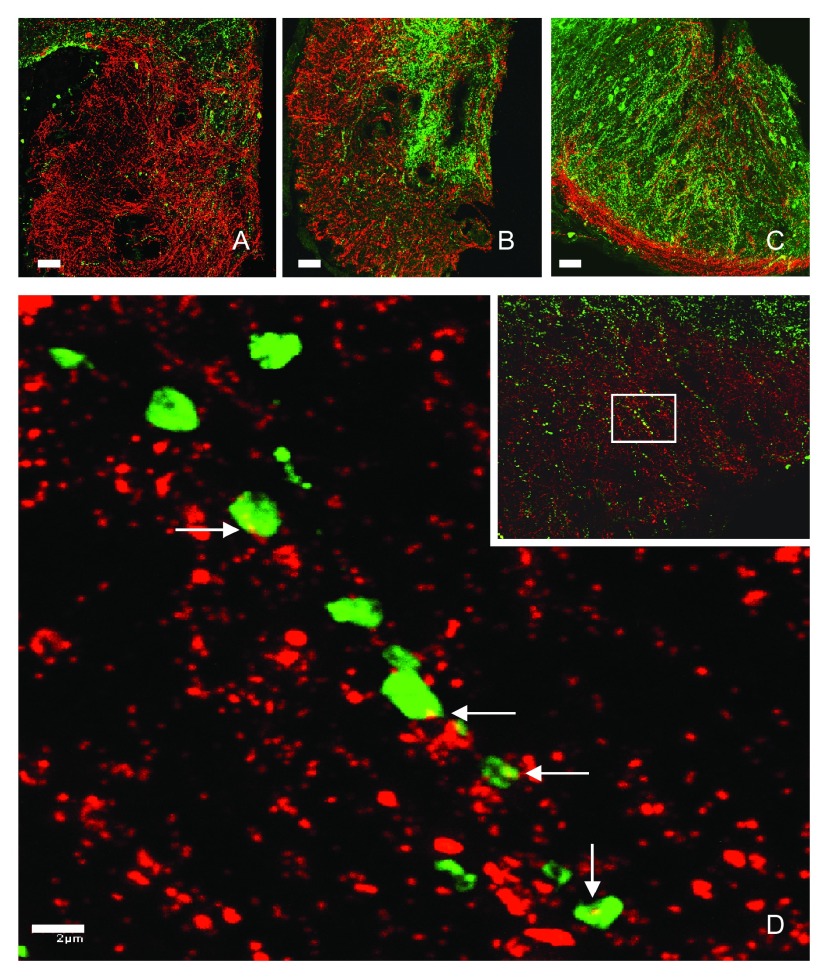
Kisspeptin projections to the median eminence intermingle intimately with GnRH fibers in the monkey. Interactions between kisspeptin (green fluorescence) and GnRH (red fluorescence) fibers in hemi-coronal sections at the anterior ( **A**), mid-tuberal ( **B**), and posterior ( **C**) aspects of the median eminence of a castrated adult male rhesus monkey. Note, in panel **B**, the heavy kisspeptin innervation of the internal zone of the median eminence and GnRH innervation of both the internal and external zones. Panel **D** shows high-power magnification of kisspeptin axonal beads in contact with GnRH fibers (white arrows) in the external zone of the median eminence shown in the inset. GnRH, gonadotropin-releasing hormone. Reproduced from Ramaswamy *et al*. ^44^ with permission of the Endocrine Society.

**Figure 4.**
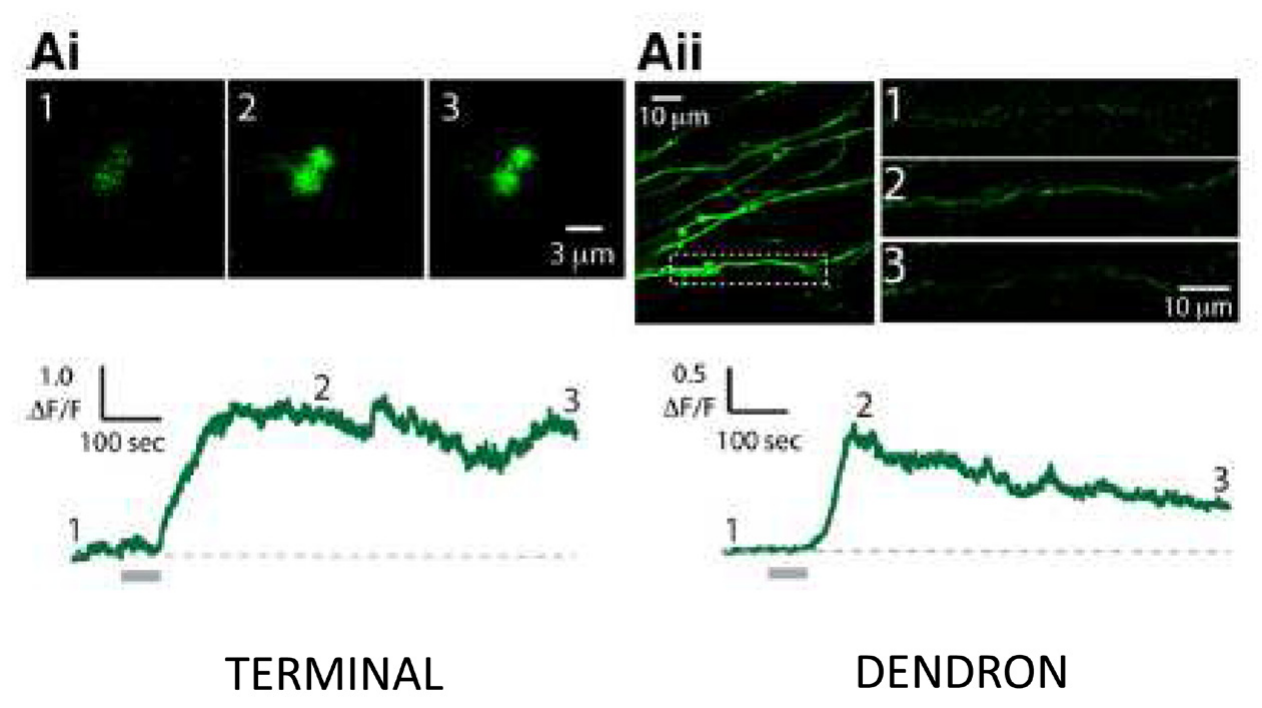
Kisspeptin elicits Ca ^++^ increases in GnRH dendrons and terminals in the mouse median eminence. One-minute discharges (“puffs”) of kisspeptin from a pipette locally applied to GnRH nerve terminals and dendrons in hypothalamic slices elicit increases in intracellular Ca ^++^ (the trigger for GnRH release) monitored by the Ca ^++^ indicator, GCaMP6 (green fluorescence), expressed in GnRH neurons of transgenic mice. Lower traces, Ca ^++^ responses to a kisspeptin puff (horizontal gray bar) on a GnRH terminal ( **Ai**) or dendron ( **Aii**). The fluorescent signals observed at times 1, 2, and 3 on the Ca ^++^ traces are shown above. ΔF/F, relative increase in fluorescence over baseline; GnRH, gonadotropin-releasing hormone. Reproduced from Iremonger *et al*. ^53^ with permission of the Society for Neuroscience.

Kisspeptin neurons in the arcuate nucleus may be distinguished from other kisspeptin neurons in the hypothalamus by the co-expression of neurokinin B and the neurokinin B receptor (TAC3R) ^45, 46^ and this feature of the arcuate cells has been exploited in studies of their function. Specifically, selective destruction of arcuate kisspeptin neurons using TAC3R-targeted uptake of saporin, a ribosome-inactivating toxin, resulted in a profound reduction in LH secretion in the ovariectomized rat ^47^, thus mimicking the effects of earlier indiscriminate neuronal destruction in the arcuate nucleus of the monkey. In addition, many kisspeptin axons and terminals in the median eminence contain neurokinin B ^48– 50^, indicating the arcuate nucleus as the origin of these projections, a finding confirmed by conditional viral tract-tracing studies in the mouse ^51^. Kisspeptin fibers and terminals are found throughout the internal zone of the median eminence ^52^, where they intermingle in an intimate fashion with GnRH projections ( Figure 3) ^44^, and kisspeptin-induced calcium currents in GnRH terminals and projections in and around the median eminence were recently described ( Figure 4) ^53^. Interestingly, studies of GnRH projections to the median eminence in the mouse indicate that they exhibit properties characteristic of both axons and dendrites, and the term dendron was coined by Herbison’s group to describe these processes ^54^ ^IV^. Such functional morphology would facilitate control of GnRH release by kisspeptin (and other neuropeptides) at the level of the median eminence. It should be noted that GnRH perikarya in the MBH in the ewe also appear to receive synaptic input from arcuate kisspeptin neurons ^55^, and the fos proto-oncogene protein (FOS, a marker of cell activation) was induced in these GnRH neurons in association with stimulation of LH pulses that followed naloxone administration ^56^.

**Figure 5.**
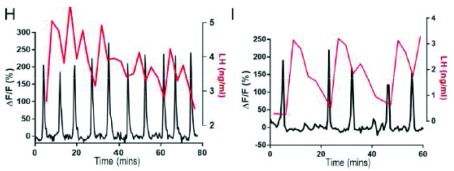
Electrophysiological activity in KNDy neurons is tightly correlated with discharges of luteinizing hormone in mouse. High-fidelity correlation between electrophysiological activity (reflected by intracellular Ca ^++^) of kisspeptin (KNDy) neurons in the arcuate nucleus of gonadectomized male mice monitored *in vivo* using an optogenetic approach (black trace) and the pulsatile secretion of luteinizing hormone (LH) (red) as tracked by measuring plasma concentrations of the gonadotropin. Reproduced from Clarkson *et al*. ^57^ with permission from the National Academy of Sciences.

Definitive evidence that kisspeptin neurons provide the output of the hypothalamic GnRH pulse generator was recently provided by Herbison’s group ^57^. Using transgenic mice in which a Ca ^++^ reporter was targeted to arcuate kisspeptin neurons, these investigators demonstrated *in vivo* that abrupt Ca ^++^ signals in these neurons, like the volleys of MUA recorded earlier in the MBH, were temporally correlated with high fidelity to pulses of LH secretion ^57^ ( Figure 5). Moreover, optogenetic activation of arcuate kisspeptin neurons *in vivo* for periods equivalent to the duration of the endogenous Ca ^++^ events elicited LH pulses comparable to those observed spontaneously, whereas optogenetic silencing resulted in a decrease in the frequency and amplitude of LH pulsatility ^57^. Termination of a brief period of optogenetic silencing resulted in an immediate discharge of LH, followed in all mice examined by a spontaneous LH pulse after a “time-locked” interval, indicating that the *Kiss1/NKB/Dyn* (KNDy) neurons are the pacemaker of the GnRH pulse generator rather than the relay of an upstream timing signal.

## Mechanism timing pulsatile arcuate kisspeptin release in the median eminence

In 2006, two groups found that neurokinin B axons and nerve terminals in the arcuate nucleus of rat co-localized dynorphin and abutted cell bodies that also co-expressed these two neuropeptides ^58, 59^. At the time, dynorphin and neurokinin B were viewed as inhibitory and stimulatory, respectively, to LH secretion. The group of Rance ^58^ also reported that arcuate dynorphin neurons expressed TAC3R and proposed that autofeedback within this population of neurons likely provides a mechanism to synchronize their activity ^58^. Three years later, Topaloglu *et al*. ^60^ reported that loss-of-function mutations of *TAC3R* or *TAC3*, the gene encoding neurokinin B, were associated with hypogonadotropic hypogonadism and delayed or absent puberty in humans; this phenotype is very similar to that reported for inactivating mutations of *KISS1R* six years earlier ^24, 25^. Excitement surrounding the finding by Topaloglu *et al*. was amplified by an earlier report that neurokinin B in sheep was expressed in the same arcuate neurons as those that synthesize kisspeptin ^46^ because this immediately led to the realization that two neuropeptides expressed by the same hypothalamic cells were both required for puberty and presumably therefore for pulsatile GnRH release ^61^. Because double-label immunohistochemistry indicated that most kisspeptin neurons in the arcuate nucleus of the ewe expressed both neurokinin B and dynorphin ^46^, it became apparent that the arcuate kisspeptin neurons and the neurokinin B/dynorphin neurons described earlier were one of a kind and these neurons were later assigned the acronym, KNDy ^62^.

The idea of autofeedback within this population of arcuate neurons was further pursued by Steiner’s group ^45^. Employing *in situ* hybridization, they showed that, in mouse, 20% of arcuate *Kiss1* neurons also express *Kor*, the gene encoding the kappa opioid receptor (KOR, a dynorphin receptor) ^V^, and proposed an early KNDy model of GnRH pulse generation ^45^. In essence, this model posited that KNDy in the arcuate nucleus possess an intrinsic tendency to activate. Activation was proposed to trigger synaptic release of neurokinin B within the nucleus, which in turn would amplify and synchronize firing by the network, resulting in the output of kisspeptin in the median eminence; this view is consistent with later findings that the action of neurokinin B to elicit GnRH-dependent LH discharges in the monkey lies upstream of KISS1R ^63^ and that intracerebro-ventricular (ICV) administration of TAC3R agonists in the rat and ewe induced the expression of FOS in KNDy neurons ^64, 65^. Dynorphin was also posited to be concomitantly released, and with a small phase lag this neuropeptide was proposed to terminate the activity of the network. Another episode of synchronized activity in the KNDy network would be triggered following the waning of the inhibitory activity of dynorphin.

Evidence that kisspeptin itself is involved in generating synchronized activity within the KNDy network is largely negative. It is now generally recognized that KNDy neurons do not express *Kiss1R*/KISS1R ^67^, and in studies of hypothalamic slices from mouse, the electrical activity of arcuate kisspeptin (KNDy) neurons expressing GFP was unaffected by kisspeptin ^68^. Similarly, kisspeptin-induced LH release in the rat and goat occurred without eliciting volleys of MUA in the arcuate nucleus ^69, 70^, which are now recognized to be generated by KNDy neurons (see below).

On the other hand, intra-arcuate injection of a KISS1R antagonist resulted in a modest slowing of LH pulse frequency in sheep and rat ^71, 72^ whereas that of kisspeptin induced an LH discharge in the rat. However, the possibility that the site of action of the KISS1R ligands administered directly to the arcuate nucleus is exerted at the level of the GnRH dendron has been raised ^40^. It will be important to clarify this uncertainty as the LH response to intra-arcuate–administered KISS1R antagonist in the absence of KISS1R expression by KNDy neurons led Goodman *et al*. ^73^ to incorporate a non-KNDy neuronal phenotype into the KNDy model of GnRH pulse generation.

An argument for a role of kisspeptin in determining the frequency of the GnRH pulse generator has also been proposed on the basis of findings that continuous infusions or a subcutaneous bolus injection of kisspeptin to healthy men and women and to women with hypothalamic amenorrhea resulted in an increase (not always statistically significant) in the number of LH discharges ^74– 79^. When evaluating these data, it should be noted that when LH levels are low, as in young gonad-intact adults, identification of LH pulses (and therefore their precision in reflecting GnRH pulse frequency) may be less reliable than in the castrate or agonadal situation where LH secretion is elevated and discharges of the gonadotropin are generally robust. Therefore, the possibility that exogenous kisspeptin provided in these clinical studies has in some cases increased the responsivity of the GnRH neuronal network to endogenous kisspeptin pulses should be considered.

The possibility that glia also comprise a component of the KNDy model of pulse generation has been proposed as a result of the *in vitro* finding that cultured GFP-expressing kisspeptin cells and neighboring glia isolated from fetal mouse hypothalamus exhibit synchronized Ca ^++^ oscillations in response to the neurokinin B agonist, Senktide ^80^.

The recent acceptance that the MUA recorded in the vicinity of the arcuate nucleus is generated by KNDy neurons ^40^, thereby confirming the view long held by some that MUA is indeed an electrophysiological property of the GnRH pulse generator, adds considerable importance to earlier goat studies that had monitored this activity while pharmacologically probing signaling by KNDy peptides within the arcuate network of KNDy neurons. Specifically, ICV injection of dynorphin to ovariectomized animals interrupted spontaneous volleys of MUA whereas administration of a KOR antagonist (nor-binaltorphimine, or nor-BNI) resulted in a marked acceleration in this electrophysiological parameter and an increase in the duration of the volley ^81, 82^ ^VI^. On the other hand, neurokinin B evoked an immediate MUA volley or train of such volleys. It is to be noted that, in the ewe, local pharmacological manipulation of KNDy signaling within the arcuate nucleus modulated pulsatile LH release in a manner generally consistent with that observed when MUA was used to monitor the GnRH pulse generator in the goat ^72, 82^. Additionally, neurokinin B and dynorphin increased and decreased, respectively, the firing of arcuate kisspeptin (KNDy) neurons in studies of hypothalamic slices in mice ^68^. Finally, ICV or IV administration of TAC3R antagonists (MRK-08 or ESN 364, respectively) in the ovariectomized ewe resulted in the interruption of pulsatile LH secretion ^82, 83^, and various parameters of LH secretion were suppressed by chronic oral administration of ESN 364 to castrate male monkeys, or MLE4901 (another TAC3R antagonist), to men and women ^84– 86^.

Data on the action of endogenously released arcuate neurokinin B and dynorphin have recently become available and are consistent with those derived from earlier classic pharmacological approaches. An elegant electrophysiological study by Kelly and Rønnekleiv *et al*. ^87^, using brain slices from transgenic mice, demonstrated that photostimulation of arcuate KNDy neurons for 10 seconds resulted, in these neurons, in the development of a slow excitatory post-synaptic potential (EPSP) with a time course of several minutes. The slow EPSP was blocked by a TACR3 antagonist, indicating that the potential is driven by neurokinin B released from KNDy cells, and presumably underlies the Ca ^++^ currents and volleys of MUA that result in the discharge of kisspeptin from KNDy terminals in the median eminence. Optogenetic activation of KNDy neurons also elicited dynorphin release as indicated by the finding that concomitant administration of a KOR antagonist potentiated the slow EPSP; the action of dynorphin was argued to be pre-synaptic because the response to an NKB agonist was not suppressed ^87^. Weems *et al*. ^88^ have also argued that dynorphin is released in the arcuate nucleus of the sheep during the generation of a GnRH pulse. Their conclusion is based on an increase in immunofluorescent-identified KOR internalization in the cell body of KNDy neurons in association with a GnRH pulse induced by ICV administration of neurokinin B. This finding would suggest a post-synaptic action of dynorphin in contrast to the pre-synaptic action earlier proposed in mice ^87^.

As the foregoing data strongly implicate a role for dynorphin in terminating the pulse of activity in KNDy neurons, the findings that, in the rat, intra-arcuate or ICV administration of nor-BNI influenced neither MUA recorded in this nucleus nor pulsatile LH secretion ^90, 91^ require an explanation. In this regard, Goodman *et al*. ^82^ suggested that there may be either a redundancy in opioid signaling underlying pulse termination in the rat or an alternative mechanism in this species.

**Figure 6.**
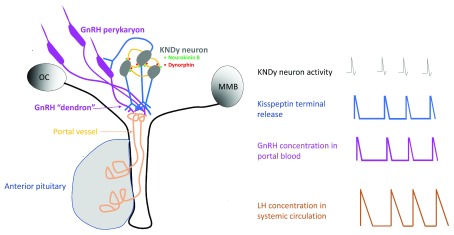
Simple schematic of the KNDy neuron model of the GnRH pulse generator. Small green and red filled circles indicate neurokinin B and dynorphin auto-synaptic release, respectively, within the arcuate nucleus. The temporal dynamics of the release of these two neuropeptides at this location are unknown. GnRH, gonadotropin-releasing hormone; MMB, mammillary body; OC, optic chiasm.

The arcuate nucleus is a bilateral structure at the base of the third ventricle, and evidence for communication between KNDy neurons on either side of the MBH, and by inference synchronization of activity, was initially provided in the rat by the finding that anterograde-labelled neurokinin B neurons on one side of the hypothalamus projected to the contralateral arcuate nucleus. In one animal close apposition between labeled neurokinin B fibers and contralateral neurokinin B perikarya was observed ^92^. The latter observation was confirmed and extended in a goat study, which also directly demonstrated synchronization of MUA monitored simultaneously in the arcuate nucleus on either side of the third ventricle ^93^. More recently, electrophysiological studies have demonstrated that photostimulation of kisspeptin fibers from cell bodies on one side of the arcuate nucleus evokes in kisspeptin neurons in the contralateral nucleus the same neurokinin B–dependent small EPSPs described above ^87^. A simple schematic of the KNDy model for GnRH pulse generation is shown in Figure 6.

## Questions that remain to be addressed

### Are the three KNDy peptides the sole interneuron signals used by the arcuate GnRH pulse generator?

Arcuate kisspeptin neurons likely release neurally active substances in addition to the KNDy peptides ^40^ and therefore it is reasonable to ask the question: do these contribute to GnRH pulse generation? One neurotransmitter of particular interest is glutamate. This is because (1) cell bodies and terminals of KNDy neurons are positive for the vesicular glutamate transporter type 2 (VGLUT2) or the mRNA encoding the protein ^55, 94, 95^, and glutamate release by arcuate KNDy neurons has been demonstrated in the mouse ^87^; (2) VGLUT2-positive terminals are in close contact with KNDy perikarya in the sheep ^55, 96^; (3) glutamate and the glutamate analog, *N*-methyl D-aspartate (NMDA), induced burst firing in arcuate kisspeptin neurons *in vitro* ^97^; (4) DL-2-amino-5-phosphonopentanoic acid, an antagonist of the ionotropic glutamate receptor known as the NMDA receptor, interrupted LH pulsatility in the castrate male rat ^98^; and (5) repetitive IV administration of NMDA, an agonist of the cognate receptor, closely mimics the GnRH-dependent LH-releasing action of intermittent kisspeptin stimulation in the juvenile male monkey model described above ^99^. The findings that peripheral administration of NMDA failed to induce LH release in mice null for *Kiss1* and *Kiss1R* ^100^ suggest that the GnRH-releasing action of NMDA may be mediated by arcuate KNDy neurons. In this regard, it is interesting to note that expression of c-FOS was reported in glutamate neurons in the arcuate nucleus of the ewe during a spontaneous GnRH pulse ^96^. However, the notion that glutamate is involved in the GnRH pulse generator is contradicted by the finding that in the ewe an intra-arcuate injection of MK801, also an NMDA receptor antagonist, failed to influence LH pulse frequency or amplitude ^72^. Clearly, the role of glutamate in GnRH pulse generation demands further exploration.

Before the advent of the kisspeptinocentric era of reproductive neuroendocrinology, the catecholamine, norepinephrine, was recognized as an important factor in the regulation of LH secretion ^101^. Nearly half a century ago, Knobil’s laboratory reported that IV administration of the alpha-adrenergic blockers, phentolamine or phenoxybenzamine, resulted in a profound interruption of pulsatile LH secretion in the ovariectomized monkey ^102^. This effect could not be attributed to a systemic hypotension or to an action of the blockers to inhibit GnRH responsiveness at the pituitary level ^103^. More recently, an action on the KNDy GnRH pulse generator was confirmed by the finding that volleys of MUA in the vicinity of the arcuate nucleus were arrested or retarded following administration of phentolamine in the ovariectomized monkey and rat ^89, 104^. These striking effects of alpha-adrenergic receptor blockers on GnRH pulse generator activity remain difficult to reconcile with the finding that surgical isolation of the MBH does not interrupt GnRH pulse generation over the long term (see above). This is because the major source of hypothalamic adrenergic tone is provided by projections from the brainstem ^101^ and therefore isolation of the MBH would be expected to greatly reduce any adrenergic input to this region of the hypothalamus and therefore mimic the effect of alpha blockers on LH secretion. As described above, this is not the case. One explanation for this paradox is that the impact of a loss in adrenergic input to the KNDy network, effected (for example) by surgical isolation of the MBH, is acute and that after an ill-defined time the KNDy network, because of plasticity posited to characterize the neural regulation of GnRH release ^101^, re-acquires the ability to generate kisspeptin pulses in the absence of a normal adrenergic input. Such a scenario would be easy to test empirically. With the extant data, however, it seems reasonable to propose that although adrenergic input may be viewed as permissive for pulse generation, it appears over the short term to be obligatory rather than modulatory and therefore examination of the neurobiology underlying the adrenergic influence on the KNDy network is of considerable importance.

Somewhat later in the pre-kisspeptin era, the laboratory of Terasawa studying gonadectomized adult rhesus monkeys described striking intermittent increments in neuropeptide Y (NPY) content in sequential perfusates of stalk median eminence (S-ME) that were concomitantly tightly correlated with discharges of GnRH ^105^. Moreover, local delivery of an NPY antibody into the S-ME resulted in a reduction in the overall content of NPY suggesting that signaling by this neuropeptide was in part responsible for the pulsatile release of GnRH. Although these findings in the monkey are unambiguous, they have been largely overlooked; perhaps because 1) mice null for NPY are fertile ^106^, and 2) the ever increasing importance that has been assigned to kisspeptin in GnRH pulse generation since 2003. Recently, however, the relationship between NPY and GnRH release described in the 1990s in the S-ME of the monkey, has been resurrected and correctly identified by Terasawa ^107^ as presenting an “unresolved question” that should be addressed when further evaluating the KNDy model for GnRH pulse generation. The presumption that NPY neurons in the arcuate neurons ^108, 109^ are the principal source of NPY in the S-ME is reasonable, but multiple mechanisms may be posited to underlie the synchrony reported for NPY and GnRH pulses in the monkey. Clearly it would be of considerable interest to determine whether this phenomenon is found in the mouse and, if so, to explore with contemporary neurobiological approaches its relationship to KNDy neuron activity.

### What is the peptide makeup of KNDy neuron terminals and what is the temporal pattern of their release from these sites?

Because KNDy neurons synthesize at least three peptides, one question that needs to be addressed is the terminal composition of their peptides and the dynamics of release both within the arcuate nucleus and at the level of GnRH processes in the median eminence. As discussed by Herbison ^40^, the dogma is that a particular neuronal phenotype releases the same set of transmitters from all of their synapses (Dale’s principal). Whether this applies to the KNDy neurons is not known. It appears unlikely that kisspeptin, if released within the arcuate nucleus along with neurokinin B and dynorphin, has a critical action on KNDy neurons (see above). On the other hand, at the level of the median eminence, dynorphin (if released) may well modulate the action of kisspeptin at the GnRH terminal, as GnRH neurons have been reported to express KOR in some species ^67^. In this regard, dynorphin release from KNDy nerve terminals onto GnRH cell bodies in the MBH of sheep was recently proposed to play a role in GnRH pulse termination in this species ^88^. The situation with TAC3R is less clear. Although this receptor has not been consistently detected in GnRH soma ^67^, GnRH processes in the median eminence of the rat co-express TAC3R ^110^, and in a study employing hypothalamic slices from mice, application to the bath of Senktide, a TAC3R agonist ^111^, elicited GnRH release from terminals in the median eminence. Therefore, neurokinin B, if released from KNDy terminals in the median eminence, is likely to modulate GnRH release. Neurokinin B is a member of the tachykinin family of peptides that include substance P and neurokinin A, and the issue of redundancy in neurokinin signaling in GnRH pulse generation, particularly in rodents, is of emerging interest. This subject was recently reviewed by Lehman and Goodman *et al*. ^72^ and will not be discussed here.

Although there is evidence from ultrastructure studies of distinct neurosecretory granules for each of the KNDy peptides ^112^, essentially nothing is known regarding the trafficking of the KNDy peptides from their site of synthesis in cell bodies in the arcuate nucleus to terminals within this nucleus and those in the median eminence.

### Is the KNDy model of GnRH pulse generation applicable to both sexes and all mammalian species?

Another question is whether the KNDy model of GnRH pulse generation may be applied universally across all mammalian species, and most importantly to humans. The cornerstone of the view that this may not be the case stems from the finding that only a small proportion of kisspeptin neurons in the infundibular (arcuate) nucleus of young men and post-menopausal women are immunopositive for dynorphin ^113, 114^. However, alternative splicing and processing of prodynorphin transcripts in the human genome are relatively complex ^115^, and detection of the encoded peptides by available antibodies may be limited. Consistent with this possibility is the finding that expression patterns of the gene that encodes dynorphin (also known as *PDYN*) in humans do not parallel those of the immunoactive protein. With *in situ* hybridization, the prodynorphin (PDYN) mRNA was detected in a large number of neurons in the infundibular nucleus in both pre- and post-menopausal human brains ^116, 117^. Moreover, the number of infundibular neurons expressing this message was smaller in the post-menopausal brain where the transcript was found in hypertrophied cell bodies: a pattern and location of expression suggesting that the PDYN mRNA-expressing neurons are indeed KNDy neurons. Thus, at present, it would appear inappropriate to reject the KNDy model of GnRH pulse generation as fundamentally applicable to all mammalian species.

Although most laboratories have employed female animals to study the KNDy model of GnRH pulse generation, available data suggest that the organizational or programming action of perinatal gonadal steroids on the arcuate population of kisspeptin neurons is less marked than that on the more anterior population of these cells in the preoptic area ^62, 118^. Therefore, it seems reasonable to propose that the neurobiological mechanisms underlying the operation of the GnRH pulse generator in the male, the electrophysiological activity (Ca ^++^ currents) of which was recently described in the male mouse ^119^, are not fundamentally different from those in the female. The somewhat sensational finding that GnRH pulse generation by a male monkey was capable of driving folliculogenesis, ovulation, and corpus luteum function in ovarian tissue transplanted subcutaneously ^120^ may reflect an absence of major sex differences in primate GnRH pulse generation.

### Can GnRH pulsatility be maintained in the absence of an intermittent kisspeptin input to the GnRH network?

An initial report that stimulated interest in this possibility was provided by Mayer and Boehm ^121^, who ablated kisspeptin neurons during early development by generating mice which expressed diphtheria A toxin in cells expressing the *Kiss1* gene. When the hypothalamus of these animals was examined on postnatal day 15, immunoactive kisspeptin neurons in the arcuate nucleus were absent. Remarkably, such female mice progressed through puberty and became fertile adults; this finding led to what might be viewed as a heretical conclusion (in light of the evidence presented above) that KNDy neurons are not essential to GnRH pulse generation. On the other hand, when kisspeptin neurons were ablated post-pubertally, ovarian cyclicity was profoundly interrupted, as would have been predicted from the KNDy model for GnRH pulse generation. The authors hypothesized that, during early development of the hypothalamus, a neuronal circuit devoid of kisspeptin neurons is formed that compensates for the ablation of KNDy neurons and drives GnRH pulsatility at and after puberty. However, there is an alternative explanation, namely that the plasticity induced by kisspeptin neuron ablation lies distal to the KNDy neuron (that is, in the GnRH neuron–pituitary–ovarian axis). This may take one of two forms. First, GnRH neurons in the absence of kisspeptin neurons might develop the intrinsic ability to generate GnRH pulses; this is not unreasonable in view of studies of GnRH neurons in culture ^17^. Second, the GnRH neuron–pituitary–ovarian axis is able to support cyclicity in the absence of pulsatility. Both of these possibilities are compatible with the concomitant finding that ablation of KISS1R cells during early development did not prevent puberty and subsequent fertility. This is because a small number of GnRH neurons were unaffected by the latter genetic strategy, and it has been recognized for many years that only a small number of GnRH neurons are required to drive the pituitary–ovarian axis ^17^. An insight into these two lines of speculation might be gained by examining moment-to-moment changes in LH concentrations in the transgenic animals of Mayer and Boehm.

Two other approaches aimed at generating an experimental model in which kisspeptin pulsatility is absent or interrupted have been employed. In the first, human subjects bearing loss-of-function mutations in either TAC3 or TAC3R and therefore lacking the neurokinin B signal posited by the KNDy model to trigger pulses of kisspeptin were studied ^122^. In such individuals, continuous IV administration of kisspeptin for 12 hours resulted in a marked increase in the number of LH pulses detected over the duration of the infusion. In the second, Clarke *et al*. ^123^, employing the ovariectomized ewe, administered a neurokinin B antagonist ICV with the aim of abolishing the intermittent kisspeptin output of the pulse generator. As was to be expected, LH pulsatility was interrupted but a continuous IV infusion of kisspeptin during the last 3 to 4 hours of neurokinin B antagonist administration precisely restored the pattern of LH pulsatility to that observed in the control period. If the pulsatile kisspeptin output to the GnRH system was indeed silenced in the foregoing models, it must be argued that a background of continuous kisspeptin signaling allows the GnRH network to pulse at a frequency identical to that which is normally dictated by pulses of kisspeptin. Although this possibility does not detract from the fundamental significance of the KNDy neurons in driving pulsatile GnRH secretion under physiological conditions, it is an unexpected result that merits confirmation and then further study because of its potential relevance to therapeutic approaches used in the infertility clinic ^123^. Additional studies that examine the action of continuous kisspeptin administration in models that employ a different approach to abrogating the intermittent kisspeptin output of the pulse generator (for example, KNDy neuron ablation or silencing) are likely to be particularly helpful. In this regard, in the anestrous ewe, pulsatile GnRH release is greatly repressed by seasonal cues, but Caraty *et al*. ^37^ were unable to induce pulsatile LH secretion in such a model by a 4-hour infusion of kisspeptin. Although there are differences between the ovine models used by the groups of Clarke ^123^ and Caraty ^37^, a potential explanation to reconcile the contradictory results is not forthcoming at present.

## Footnotes

I. As was noted by Knobil in 1992 ^7^, the pulsatile nature of gonadotropin secretion was also reported in 1970 by a group of French investigators studying men and women ^8^ but this clinical observation commanded little attention from the neuroendocrine community.

II. A pulsatile pattern in GnRH concentrations in hypophysial portal blood had been reported earlier for the hypophysectomized monkey and rat by Carmel *et al*. ^21^ and Sarkar and Fink ^22^, respectively. However, the experimental models employed by these investigators prevented correlation of GnRH levels with episodic LH release.

III. Pioneering studies of MUA in the MBH were initiated by Knobil’s laboratory in the latter half of the 1970s ^23^.

IV. To date, the anatomical structure termed “dendron” has not been described for species other than mouse.

V. A more recent study of ovine and rat hypothalamus employing dual-label immunofluorescence revealed a much higher co-localization of KOR and kisspeptin in neurons in the arcuate nucleus ^66^.

VI. Earlier, Nishihara *et al*. ^89^ had found that the frequency of MUA volleys in rat was accelerated following administration of naloxone, a non-selective opioid antagonist.
